# Ultra-Processed Food Consumption, Additive Co-occurrence, and Associated Risk Factors Among University Students in Peru

**DOI:** 10.12688/f1000research.166729.2

**Published:** 2026-02-14

**Authors:** JHOSELINE STAYCE GUILLEN SANCHEZ, Mónica Saenz Tolay, Luis Cabanillas Chirinos, Ana María Pita Ruiz

**Affiliations:** 1 Universidad Cesar Vallejo, Trujillo, La Libertad, Peru; 2 State University of Campinas, Campinas, State of São Paulo, Brazil

**Keywords:** Ultra processed foods, Food additives, Dietary intake, Public health nutrition

## Abstract

**Background:**

The rising consumption of ultra-processed foods (UPFs) is a global concern, given their poor nutritional quality and the presence of multiple additives that may pose health risks when consumed in excess. Young populations are particularly vulnerable due to lifestyle habits and the widespread availability and accessibility of these products. The present study aimed to assess the consumption of ultra-processed foods (UPFs), taking into account the co-occurrence of additives and their association with risk factors in a young population.

**Methods:**

A two-phase cross-sectional study was conducted. In the first phase, 500 ultra-processed food products available on the Peruvian market were analysed and classified into five groups: meat products, cereals, confectionery, dairy, and beverages, in order to identify the presence and frequency of additives. In the second phase, a structured survey was administered to 385 students, collecting data on UPF consumption over the previous 24 hours, along with variables related to lifestyle. Chi-square tests and ordinal logistic regression were used to analyse the associations.

**Results:**

The categories with the highest levels of consumption were carbonated beverages (59.6%), meat products (54.7%), and dairy in various forms (66.3%). A high frequency of co-occurrence was identified between colourings and acidulants, particularly INS 133, INS 150d, and INS 339i. Statistical analysis revealed a significant association between UPF consumption and male sex (OR = 1.603), as well as alcohol consumption (OR = 2.059), while age showed an inverse relationship (OR = 0.756). Other variables, such as physical activity and smoking, did not demonstrate a significant association.

**Conclusion:**

It is concluded that excessive UPF consumption varies according to age and sex and represents a continuous source of exposure to food additives. In addition, a statistically significant association was observed between UPF consumption and alcohol consumption, which may indicate a relationship of relevance from a public health perspective; however, a causal relationship cannot be established.

## Introduction

Ultra-processed foods (UPFs), pre-packaged products predominantly manufactured on an industrial scale, have come to dominate the food environment in both high- and low-income settings ( Baric et al., 2025). In Latin America, their consumption has surged in recent years, particularly in the form of beverages. Uruguay has experienced a 146% increase in UPF sales, followed by Bolivia (130%) and Peru (107%). Similarly, Brazil and Peru have witnessed a 38.9% rise in fast food intake, often replacing more nutritious traditional diets ( Instituto Nacional de Salud, 2023).

In Peru, Choque-Quispe et al. (2023) reported that 3.9% of adolescents aged 15 to 18 consume UPFs daily, while 75.5% consume them one to three times per month. Beverages were the most frequently consumed (30.4%), followed by processed meats and sweets (both 17.6%), and dairy products (12.7%). In Chile, students aged 17 to 24 showed higher consumption rates of sauces and spreads (78.4%), processed meats (76.3%), and beverages (74.8%), whereas intake of fast foods (28.1%), savoury snacks (35.3%), and cereal-based items (37.4%) was comparatively lower ( Vilugrón et al., 2022). Among older adults (mean age: 47), UPF consumption was notably lower (24%), with a marked preference for minimally processed foods (51.6%) ( Anjos et al., 2024). In Spain, Gearhardt et al. (2023) identified signs of UPF addiction in 14% of the adult population and 12% of children, characterised by impulsive behaviour, emotional dysregulation, and diminished quality of life.

UPFs can pose significant health risks when consumed excessively or without adherence to age-specific dietary recommendations. These products typically contain food additives, substances deliberately introduced to alter physical, chemical, biological, or sensory properties, predominantly used for technical purposes in industrial manufacturing ( World Health Organization [WHO], 2023). They also tend to be rich in saturated fats, sugars, and sodium, all linked to an elevated risk of non-communicable diseases, including cancer, diabetes, and cardiovascular conditions. Notably, approximately 72% of daily sodium intake originates from added salt and food additives, with the remainder derived from natural sources (8%) and table salt (20%) ( Carbajal & Moreno, 2023; Dicken et al., 2024; Lou et al., 2021). Furthermore, sugar-sweetened beverages have been associated with increased body weight, type 2 diabetes, and non-alcoholic fatty liver disease ( Malik & Hu, 2022).

The widespread shift from home-cooked, natural foods to ready-to-eat UPFs represents a broader transformation in dietary practices ( Aracta-Maquera, 2023; Choque-Quispe et al., 2023). These products—from soups and fizzy drinks to processed meats—are readily available, affordable, and increasingly perceived as convenient staples. However, their health consequences are often overlooked, despite regulatory efforts such as the Peruvian Healthy Eating Promotion Law for Children and Adolescents ( Ley 30021, Congreso de la República del Perú, 2013), which mandates clear front-of-pack labelling for excessive contents of sodium, sugars, and saturated fats ( FAO, 2024a; Avilés et al., 2017).

Generation Z, born between approximately 1995 and 2010, has matured within a highly digitised environment and faces challenges including nutritional autonomy, financial constraints, academic demands, and limited experience with meal planning. These conditions often lead to choices that prioritise convenience and visual appeal over nutritional value. Wanjohi et al. (2025) found that this demographic tends to view UPFs as modern, urban, and desirable, whereas traditional foods are often regarded as outdated or unappealing.

Diet remains a modifiable risk factor, and the presence of food additives may exacerbate health risks. Emulsifying polysaccharides, for example, have been implicated in inflammatory bowel diseases ( Nickerson et al., 2015; Laudisi et al., 2019). Mono- and diglycerides of fatty acids (INS 471 and INS 472) have been linked to increased cardiovascular risk ( Sellem et al., 2023), while sweeteners such as aspartame and acesulfame-K are under scrutiny for their potential carcinogenicity ( Debras et al., 2022). A systematic review of cohort studies found associations between non-nutritive sweeteners—including aspartame, sucralose, and steviol glycosides—and increased body weight, waist circumference, obesity, hypertension, and metabolic syndrome ( Azad et al., 2017).

In Peru, the General Directorate of Environmental Health and Food Safety (DIGESA), under the Codex Alimentarius framework, is responsible for enforcing these regulations. Its mandate includes preserving food quality, improving shelf stability, and facilitating processes such as manufacturing, preparation, packaging, and storage—provided that additives are not used to mask inferior raw materials or substandard practices ( FAO, 2024a). Nonetheless, there remains a lack of targeted research on consumption patterns, risk perception, and additive exposure among Generation Z university students, a cohort particularly vulnerable to modern food environments and autonomous dietary choices ( Barroso, 2020). This gap represents a barrier to designing effective interventions, particularly in light of the rising incidence of non-communicable diseases at increasingly early ages ( Fedde et al., 2025). Addressing these factors is essential if public health systems are to succeed in promoting healthy lifestyles.

Accordingly, the present study aims to assess the consumption of ultra-processed foods among young individuals, with a particular focus on the co-occurrence of additives and their association with health-related risk factors.

## Methods

### Research design

The study employed a cross-sectional observational design and was conducted in two phases, generating a complementary dataset.

(1) A descriptive analysis was undertaken of 500 ultra-processed food products available in major supermarket chains across Peru. The products were classified into five categories: meat products, cereals, confectionery, dairy, and beverages. Ingredient labels were examined to identify the presence and frequency of food additives, including colourings, acidulants, preservatives, antioxidants, and sweeteners Organización Panamericana de la Salud, 2015; FAO, 2024b). Product selection was carried out using non-probability convenience sampling, based on the accessibility and proximity of formal retail outlets (supermarkets) most frequently visited by the urban population. This strategy allowed for the inclusion of readily available and easily identifiable products from high-turnover supermarkets, thereby facilitating the data collection process. Nevertheless, this type of sampling may limit the representativeness of products consumed in other contexts, such as rural areas or informal markets.
(2) A structured survey was administered to a probabilistic sample of 385 university students from Generation Z (born between 1995 and 2010). The sample size was calculated using the formula for infinite populations ( Aguilar-Barojas, 2005), with a 95% confidence level, a 5% margin of error, and an expected proportion of 50%.

The data collection instrument was previously validated by three experts in food technology, nutrition, and public health to ensure its relevance and clarity. To assess its reliability, a pilot test was conducted with 20 students to evaluate the comprehensibility and consistency of the questions. This process enabled necessary adjustments to be made prior to its final administration. Both the validity and reliability coefficients of the instrument exceeded 0.7.

This study complied with established ethical standards to safeguard the rights and dignity of all participants. Informed consent was obtained voluntarily and in written form via an initial question embedded in the online questionnaire, which allowed individuals to either confirm or decline their participation. Only those who provided explicit consent were permitted to proceed. All participants were adults and received comprehensive information regarding the study’s objectives. Data confidentiality was upheld through anonymisation and the secure storage of all collected information, in strict accordance with the ethical principles outlined in the Declaration of Helsinki and the Code of Ethics of César Vallejo University, as endorsed by University Council Resolution No. 0126-2017-UCV. Participants were assured of their right to withdraw from the study at any stage without incurring any negative repercussions.

### Classification and co-occurrence of additives in ultra-processed foods

Ultra-processed foods (UPFs) were defined as industrial formulations composed of substances extracted from foods, synthetic constituents, or food additives, including acidity regulators, stabilisers, antioxidants, preservatives, and sweeteners.

A total of 500 UPF products available in the leading supermarket chains in Peru were acquired for analysis. Ultra-processed beverages were classified according to the criteria established by the Pan American Health Organization ( PAHO, 2015), which include carbonated drinks, fruit juices, sports and energy drinks, as well as ready-to-drink tea or coffee. Meanwhile, food items were categorised following the Food and Agriculture Organization ( FAO, 2023) guidelines into meat products, cereals or cereal-based products, dairy products, and confectionery.

Non-perishable beverages and foods were stored in a cool environment, whereas perishable items were kept refrigerated at 4°C until analysis. Additive identification was based on the review of ingredient lists as declared on the nutritional labels of each product. The percentage of products within each category containing at least one additive was calculated according to the methodology proposed by Lorenzoni et al. (2021), and additive groupings were determined through cluster analysis, represented using dendrograms.

### Risk factors associated with the consumption of ultra-processed foods and beverages among university students

University students were selected as the target population, as they are considered one of the primary consumer groups of ultra-processed foods due to factors such as ease of access, habitual meal-skipping, use of discretionary funds, exposure to advertising, and the influence of social media. To assess consumption, a structured questionnaire was employed, focusing on the UPFs consumed within the previous 24 hours. It is worth noting that, although this self-reported questionnaire may be subject to recall bias, this method was chosen due to its widespread use in public health and its effectiveness in estimating recent food intake. To reduce such bias, the instrument was validated by experts and pilot-tested. As noted by Martínez et al. (2022), 24-hour dietary recalls remain valid and widely accepted tools for assessing ultra-processed food consumption in the general population. The instrument comprised 19 items, including questions on ultra-processed foods and beverages, classified into five categories. A total of 329 complete questionnaires were collected, resulting in a 100% response rate. Consumption questions by product group were multiple-choice, allowing participants to select one, several, or no options. Consequently, percentages were calculated based on the valid responses within each group, which may introduce partial non-response bias. The data collected were subsequently organised, with illegible or inconsistent responses excluded to maintain data integrity.

Consumption within each food group was recorded using dichotomous responses (yes/no). A consumption score was calculated by summing the number of food groups reported as consumed the previous day, resulting in a range from 1 to 15. These scores were then grouped into three categories: 0–4, 5–10, and over 10, with scores of 5 or more considered indicative of excessive consumption ( Costa et al., 2021).

Given that food additives are subject to varying recommendations and usage limits, the five most frequently consumed UPF products were initially selected. The ingredient lists reported on their labels were then analysed to determine the quantity per serving and to identify any potential adverse effects associated with their intake.

In addition, four major modifiable risk factors associated with the development of non-communicable chronic diseases were evaluated: excessive alcohol consumption, physical inactivity, tobacco use, and poor dietary quality. Excessive alcohol intake was assessed with the question: “In the past 30 days, have you ever consumed five or more alcoholic beverages in one occasion?” Physical activity was measured by asking: “Do you engage in exercise at least once per week?”

Dietary quality was estimated based on UPF intake, with consumption of more than five UPF items on the previous day categorised as excessive.

• **Independent variable:** Level of consumption (high – medium – low)
• **Dependent variables:** Risk factors (alcohol consumption, place of food intake [home or boarding house], smoking habits, physical activity, age, and sex)

### Data analysis

A descriptive analysis was conducted using absolute and relative frequency distributions to determine the consumption of ultra-processed foods (UPFs) within the study sample. Additionally, a clustering analysis was performed using a hierarchical organisation technique to identify patterns of co-occurrence among food additives.

Inferential analysis included the chi-square test of independence, as well as an ordinal logistic regression, aimed at evaluating the impact of independent variables on the dependent variable. A significance level of 5% was adopted. For the ordinal regression analysis, 329 cases with complete data for all variables included in the model were considered. Data processing and analysis were carried out using SPSS software, version 26 (IBM).

## Results

### Clustering of food additives and UPF consumption by category

Within the category of meat products, sausages exhibited the highest consumption frequency, reaching 54.7%. Among beverages, carbonated soft drinks stood out at 59.6%. In the cereal group, pasta and noodles were consumed by 48.6% of the students. Regarding confectionery items, biscuits ranked first with 48.9%, while in the dairy category, milk in its various forms was the most preferred, with a consumption rate of 66.3% ( Table 1).

**Table 1.** Consumption patterns of ultra-processed beverages and products by category among university students, January – April 2024.

Group	Product	n°	Frequency (%)
Meat	Types of sausages ^1^	180	54.7
	Hamburger	137	41.6
	Processed meats ^2^	107	32.5
	Pork cracklings/Bacon	85	25.8
	Nuggets	47	14.3
	Pâté	6	1.8
	N/A (Not available)	93	28.3
Beverages	Soft drinks	196	59.6
	Packaged nectars/Industrialized juices	133	40.4
	Sports drinks/Energy drinks	129	39.2
	Flavored water	61	18.5
	Bottled teas	49	14.9
	None of the above	58	17.6
Cereal-based products	Pasta/Noodles	160	48.6
	Salty snacks	139	42.2
	Instant mixes	62	18.8
	None of the above	90	27.4
Confectionery products	Cookies	161	48.9
	Chocolates	150	45.6
	Pastries ^3^	121	36.8
	Hard/Chewy candies ^4^	143	43.5
	None of the above	57	17.3
Dairy products	Milk ^5^	218	66.3
	Yogurt	180	54.7
	Cheese	139	42.2
	Soy milk	26	7.9
	Flavored dairy beverages	15	4.6
	None of the above	59	17.9

*Percentages were calculated based on the total number of valid responses within each product group, rather than the total sample (n = 385), as participants could select multiple options or leave categories unanswered.

^1^ Hot dog, sausage, chorizo.

^2^ Luncheon meat, mortadella, ham.

^3^ Cakes, sponge cakes.

^4^ Gummy candies, chewing gum, lollipops, hard candies.

^5^ Milk: powdered, evaporated, condensed, cream-based.

Figure 1 illustrates how certain beverages (fruit-based, energy, and carbonated drinks) tend to cluster based on the colourings and acidity regulators they contain. In fruit-flavoured beverages, colourings INS 133 (Brilliant Blue FCF) and INS 150d (Caramel IV) were frequently found together ( Figure 1A). In contrast, energy and carbonated drinks were more likely to contain other colourings such as INS 160 (Carotene), INS 170 (Calcium Carbonate), INS 132 (Indigotine), and INS 131 (Patent Blue V) ( Figure 1B). Notably, when a product contained INS 160, it was commonly paired with INS 170, suggesting a frequent co-usage pattern. Similarly, in fruit-based drinks, the combination of INS 110 (Sunset Yellow FCF) and INS 160 was prevalent, while the most common pairing in energy and fizzy drinks was INS 110 and INS 150. However, these colourings were generally used independently of other additives. Colouring INS 102 (Tartrazine) appeared isolated in the dendrogram, suggesting relatively limited use in the analysed samples.

**Figure 1.**
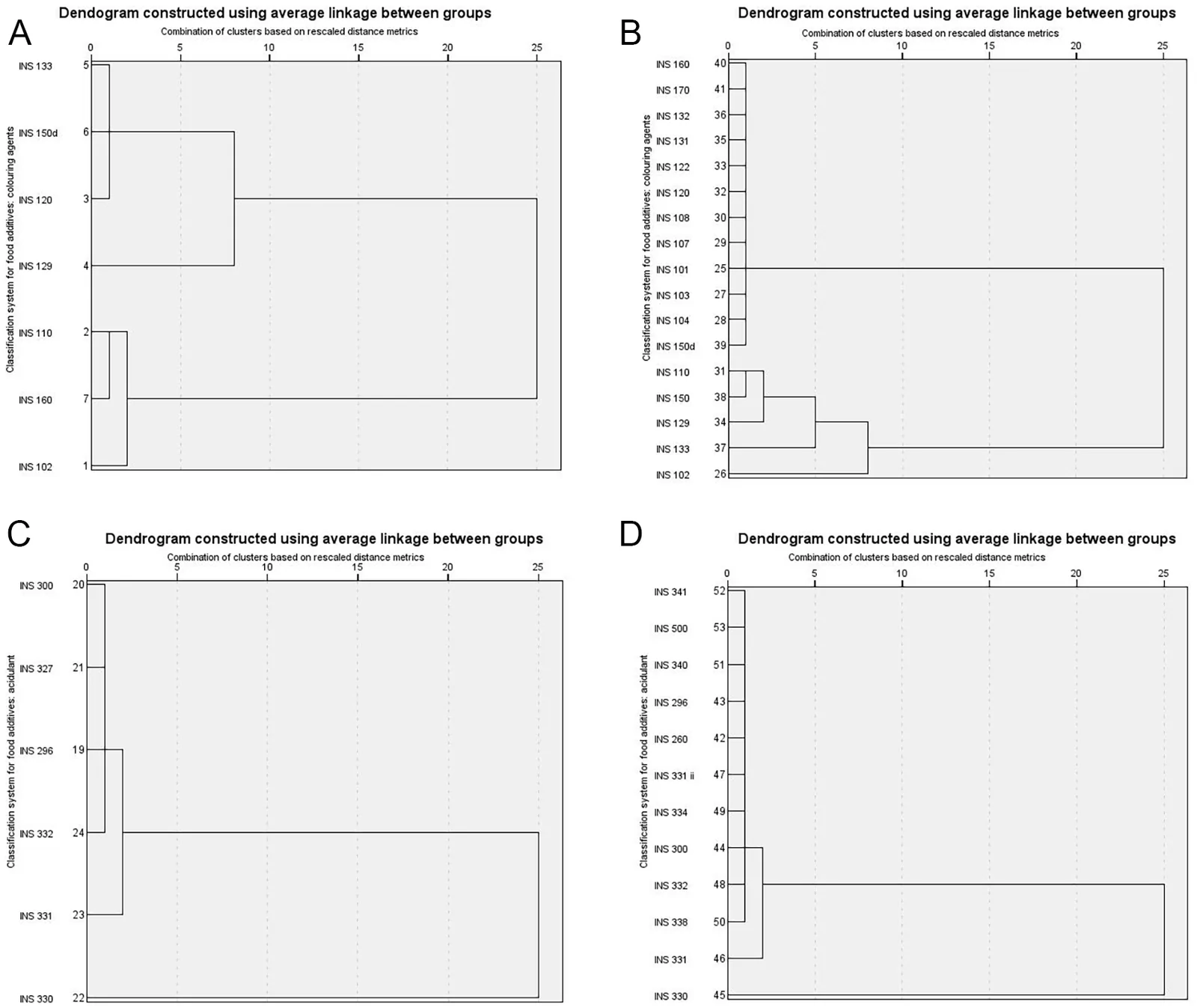
(A) Dendrogram of the most frequently used food colorants in fruit-based beverages, (B) Dendrogram of the most frequently used food colorants in energy and carbonated beverages, (C) Dendrogram of the most frequently used acidity regulators in fruit-based beverages, (D) Dendrogram of the most frequently used acidity regulators in energy and carbonated beverages.

Regarding acidity regulators, fruit-flavoured beverages frequently contained both INS 300 (Ascorbic Acid) and INS 327 (Sodium Lactate) ( Figure 1C). In contrast, energy and carbonated drinks commonly featured INS 341 (Calcium Phosphates), INS 500 (Sodium Carbonates), INS 340 (Potassium Phosphates), and INS 296 (Malic Acid). One particular acidulant showed a clustering distance nearly 25 units greater than the others ( Figure 1D), indicating infrequent co-use with other additives.

Dairy products were also clustered based on their acidity regulators, while meat products and oils and fats were grouped according to their antioxidant content. The commonly identified acidulants included INS 339i (Monosodium Phosphate), INS 452i (Sodium Polyphosphate), INS 331i (Monosodium Citrate), and INS 500i (Sodium Carbonate), the latter also appearing in carbonated and energy drinks ( Figure 2A).

**Figure 2.**
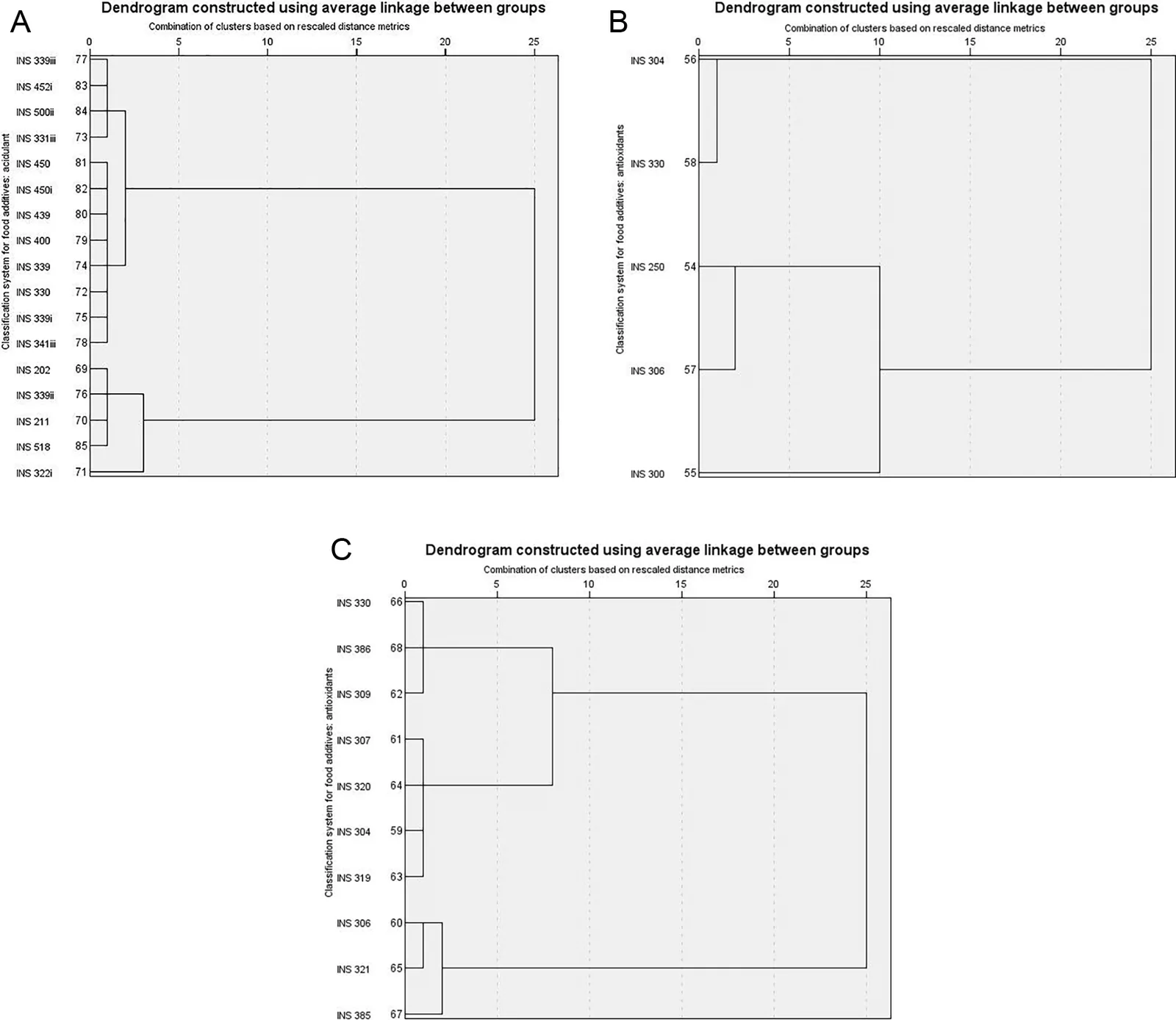
(A) Dendrogram of the most frequently used acidity regulators in dairy products, (B) Dendrogram of the most frequently used food antioxidants and preservatives in meat products, (C) Dendrogram of the most frequently used food antioxidants in oils and fats.

The antioxidant INS 330 (Citric Acid) was frequently found in both meat products and oils and fats. However, in the former, it was most often co-present with INS 304 (Ascorbyl Palmitate), while in the latter it was more commonly associated with INS 386 (Disodium EDTA). The antioxidants showing the greatest dissimilarity or least frequent use in these products were INS 300 (Ascorbic Acid) in meat products and INS 385 (Calcium Disodium EDTA) in oils and fats, respectively ( Figures 2B and 2C).

### Prevalence of risk factors in relation to the consumption of ultra-processed foods

Using the chi-square test of independence ( Table 2), a significant association was found between the consumption of ultra-processed foods and the sex of the students (p = 0.028), as well as their alcohol consumption (p = 0.005). An ordinal logistic regression model was applied to evaluate the effect of the independent variables on the likelihood of UPF consumption within the categories: Permitted, Excess I, and Excess II. The model was statistically significant (χ ^2^ = 27.082, p = 0.003), although it accounted for only 8.9% (R ^2^ = 0.089) of the variance in the dependent variable categories. The results for predictive values and Odds Ratios are presented in Table 3.

**Table 2.** Chi-Square test of independence between study variables and ultra-processed food consumption among surveyed university students (January – April 2024).

Risk factors		Ultra-processed food consumption			Statistic χ ^2^	Value “P”
		Permitted	Excess I	Excess II		
Age	16-21	48	94	68	10.466	0.106
	22-27	21	35	32		
	28-33	8	10	1		
	≥ 34	5	3	4		
Sex	Female	48	82	77	7.184	0.028*
	Male	34	60	28		
Study modality	In-person	37	61	47	2.891	0.576
	Virtual	0	5	3		
	Both	45	76	55		
Diet	At home	70	113	82	1.703	0.427
	In Student Boarding Houses	12	29	23		
Drinks alcoholic beverages	Yes	48	79	37	14.938	0.005*
	Occasionally	32	55	58		
	No	2	8	10		
Drank alcoholic beverages in the last month	Yes	69	113	80	1.798	0.407
	No	13	29	25		
Exercise/played sports in the last 3 months	Yes	62	98	73	1.220	0.543
	No	20	44	32		
Exercise at least once a week	Yes	50	81	57	0.842	0.656
	No	32	61	48		
Smokes	Yes	79	130	97	1.930	0.381
	No	3	12	8		
Total		82	142	105		

**Table 3.** Logistic regression model for the effect of risk factors on the consumption of ultra-processed foods among surveyed university students, January – April 2024.

Risk factors	B (DE)	p	95% CI for OR		
			Odds Ratio	Low	High
Age group	-0.279 (0.1446)	0.048	0.756	0.570	1.004
Sex	0.472 (0.2246)	0.036	1.603	1.032	2.490
Study Modality	-0.045 (0.2182)	0.837			
Diet	-0.307 (0.2628)	0.248			
Drinks alcoholic beverages	0.722 (0.2071)	0.000	2.059	1.372	3.089
Drank alcoholic beverages in the last month	0.001 (0.3078)	0.998			
Exercise/played sports in the last 3 months	-0.176 (0.2923)	0.544			
Exercise at least once a week	-0.082 (0.2625)	0.753			
Smokes	0.072 (0.4430)	0.874			

Specifically, male students were 1.603 times more likely to report higher UPF consumption. Additionally, for each one-point increase in alcohol consumption, the likelihood of higher UPF intake increased by 2.059 times. Conversely, for each increase in age group, students were 0.756 times less likely to report high UPF consumption. The remaining study variables showed no statistically significant effect on the outcome variable (p > 0.05) ( Table 3).

A multivariate ordinal logistic regression model was conducted to identify factors associated with UPF consumption and to assess whether these associations remained after accounting for other potentially influential variables (confounders) ( Table 4). Regarding age, participants aged 16–21 (Group 1) and 22–27 (Group 2) exhibited a higher likelihood of being in a higher UPF consumption category compared to those aged 34 and over (reference group). In contrast, those aged 28–33 (Group 3) showed a lower likelihood of high consumption. However, none of these age-related associations reached statistical significance.

**Table 4.** Ordinal logistic regression model for the level of consumption of ultra-foods among surveyed university students, January – April 2024.

	Estimate ^b^	Std. Error	Wald	Sig.	95% Confidence Interval (CI)	
					Lower limit	Upper limit
Permitted Consumption	-2.053	0.818	6.293	0.012	-3.657	-0.449
Excessive I Consumption	-0.046	0.811	0.003	0.955	-1.635	1.543
Age 16-21	0.446	0.565	0.622	0.430	-0.662	1.553
Age 22-27	0.409	0.590	0.480	0.488	-0.747	1.565
Age 28-33	-0.826	0.716	1.331	0.249	-2.229	0.577
Female	0.439	0.229	3.667	0.055	-0.010	0.887
In-person modality	-0.058	0.221	0.069	0.793	-0.490	0.375
Online modality	0.756	0.718	1.107	0.293	-0.652	2.164
Eats at home	-0.301	0.267	1.272	0.259	-0.825	0.222
Does not consume alcoholic beverages	-1.392	0.561	6.162	0.013	-2.490	-0.293
Occasional alcohol consumption	-0.630	0.532	1.403	0.236	-1.673	0.413
No alcohol in the past month	0.007	0.307	0.001	0.982	-0.594	0.608
Exercised in the last 3 months	-0.164	0.292	0.315	0.574	-0.737	0.409
Exercises once a week	-0.087	0.263	0.110	0.740	-0.602	0.427
Does not smoke	-0.098	0.492	0.039	0.843	-1.061	0.866

With respect to alcohol consumption in the past month, students who reported not drinking showed a slightly higher probability of high UPF consumption compared to those who did consume alcohol, although this difference was also not statistically significant. Based on the results from the sample of 329 students, the ordinal regression model identified a statistically significant difference in the intercept for the “Permitted” consumption level (p = 0.012 < 0.05), but not for the “Excessive I” level (p = 0.955 > 0.05), when compared to the intercept for the “Excessive II” level under the logit link function. Furthermore, the predictor “non-consumption of alcoholic beverages” had a negative and statistically significant effect (coefficient = –1.392, p = 0.013), indicating a lower likelihood of being in a higher UPF consumption level compared to those who reported alcohol consumption ( Table 5).

**Table 5.** Forecasting the level of consumption of ultra-processed foods among university students surveyed using the ordinal regression model, January – April 2024.

	Observed level	Predicted level			Total	Accuracy (%)
		Permitted	Excessive I	Excessive II		
Ultra-Processed Food Consumption	Permitted	9	62	11	82	11.0
	Excessive I	8	112	22	142	78.9
	Excessive II	1	67	37	105	35.2
	Total	18	241	70	329	48.0

## Discussion

Carbonated soft drinks were the most preferred ultra-processed beverages, consumed by over 50% of the study population, whose mean age was 24.5 ± 0.01 years. Among solid products, between 41.6% and 66.4% of students reported consumption of items in the meat, dairy, and confectionery groups (including biscuits). These high levels of intake are consistent with the findings of Mamani-Urrutia et al. (2021), who reported elevated consumption of biscuits (56%) and dairy products (54.3%) among university students. A similar order of preference was observed by Choque-Quispe et al. (2023) in Peruvian adolescents aged 14 to 18 years; however, the reported values were lower: beverages (30.4%), meat products (17.4%), and confectionery (17.4%). In Brazil, according to Simões et al. (2020), the highest UPF consumption among adolescents aged 18 to 19 in the state of Maranhão corresponded to the cereal group, particularly biscuits, cakes, and industrially processed white bread (14.1%). These patterns may be attributed to the academic demands faced by young individuals, often reflected in meal-skipping behaviours, particularly breakfast ( Maza-Ávila et al., 2022).

Milk, with a consumption rate of 66.3%, was the most consumed UPF in the evaluated population, exceeding the figures reported by Rojas et al. (2011), who found milk consumption at 42.5% and cheese at 53.8% among children aged 8.73 years. According to the National Institute of Health (2023), frequent dairy intake ensures adequate calcium supply, as milk and yoghurt have high bioavailability of this mineral. However, it is important to consider age-specific intake recommendations: 1300 mg for adolescents aged 14 to 18, and 1000 mg for adults aged 19 to 50 and children aged 4 to 8. Although milk consumption was high, so too was that of soft drinks, which may be counterproductive given that both contain sugars, increasing caloric intake without significant nutritional benefit. Moreover, high-phosphorus diets can reduce calcium bioavailability ( Takeda et al., 2014).

A total of 54.7% of participants reported sausage consumption—higher than that observed by Kotopoulou et al. (2022) in a Greek population, where sausage consumption stood at 23.8%, although a high intake of pork (41.5%) and turkey-based (32.7%) processed meats was reported. In a study by Wang et al. (2022) in Canada, bisphenol A was detected in meat products, with the highest levels found in roasted beef (118.23 ng/g), followed by cured pork (0.14 ng/g), and cold cuts such as ham, mortadella, and sausages (0.18 ng/g). Notably, these products often contain additives such as nitrites, whose excessive intake may exceed the acceptable daily intake (ADI) of 0.07 mg/kg body weight/day, posing a health risk.

Dendrogram analysis confirmed that the main antioxidants and preservatives used in meat products sold in Peru were ascorbyl palmitate (INS 304) and sodium nitrite (INS 250), respectively. Citric acid (INS 330) was identified as a common antioxidant in meat products, oils, and fats. Additionally, the most frequently used acidulants in dairy products and beverages were INS 339i, INS 452i, INS 300, and INS 341. These findings are in line with Chazelas et al. (2020), who reported that the most frequent preservatives in French meat products were sodium nitrite (INS 250), potassium nitrate (INS 252), and sodium acetate (INS 262), with sodium lactate (INS 325) and INS 262 frequently co-occurring as antioxidant and preservative, respectively.

In the confectionery group, the most commonly used colourants in combination were Allura Red AC (INS 129), Tartrazine (INS 102), Sunset Yellow FCF (INS 110), Brilliant Blue FCF (INS 133), Indigotine (INS 132), and Titanium Dioxide (INS 171), while Carmine (INS 120) was less frequently used. This pattern was also observed in the analysed beverages, except for Tartrazine (INS 102), which appeared less often. It is important to note that some of these additives are banned in certain countries due to potential adverse health effects. For example, INS 133 is banned in China, and INS 110 in Norway, Sweden, and Finland ( Vázquez et al., 2016). Furthermore, phosphates such as INS 340 and INS 341 require monitoring in individuals with kidney disease ( Garcia et al., 2017). Although these additives are not consumed directly, their presence in food implies indirect intake, which should remain within the limits established by rigorous scientific evaluations, including biochemical and toxicological studies, to determine their ADI ( WHO, 2023).

An analysis based on data from the “Pesquisa de Orçamentos Familiares” for the years 2008 and 2018 revealed that UPFs accounted for 19.7% of total caloric intake during 2017–2018. The study reported higher consumption among women than men ( Louzada et al., 2018) and found an association between UPF-rich diets and an unfavourable cardiometabolic risk profile, which increases the likelihood of developing cardiovascular disease ( Pagliai et al., 2021).

The results of the statistical analysis indicate that certain individual factors are associated with higher consumption of ultra-processed foods (UPF). According to the logistic regression model, being male was associated with a 1.603 times greater likelihood of high UPF consumption (p = 0.036). Similarly, alcohol consumption was significantly associated with a higher level of UPF intake (OR = 2.059; p < 0.001), even after adjusting for other sociodemographic and behavioural variables. A possible mechanism hypothesis is that both behaviours may be influenced by shared behavioural factors such as academic stress, exposure to marketing of unhealthy products, and socialisation patterns, all of which may encourage the selection of fast and easily accessible options such as UPF and alcohol, particularly among young people.

Although the ordinal regression model confirmed similar trends namely, a lower probability of high consumption among women and individuals who do not consume alcohol other variables, including age, study modality, eating location, and physical activity, did not reach statistical significance. In terms of predictive capacity, the model was more accurate in classifying the “Excessive I” level (78.9%), but less accurate for the “Permitted” (11.0%) and “Excessive II” (35.2%) levels, yielding an overall accuracy of 48%. While the cross-sectional design limits causal inference, these results help identify relevant risk profiles and provide valuable evidence to inform public health interventions.

Excessive alcohol consumption also negatively affects dietary patterns and nutritional status. Alcohol abuse has been linked to deficiencies in essential micronutrients such as thiamine, riboflavin, niacin, pyridoxine, folic acid, vitamins A, C, D, E, and K, as well as magnesium, selenium, and zinc ( Jeynes & Gibson, 2017). Alcohol also increases the preference for foods high in sugar and fat ( Jeynes & Gibson, 2017; Schrieks et al., 2015). In a randomised clinical trial involving healthy men in the Netherlands, moderate alcohol intake was shown to increase spontaneous consumption of high-fat foods, such as pâté and salami ( Schrieks et al., 2015).

In New Zealand, Parackal et al. (2020) found that women who consumed more than four alcoholic drinks on a typical day at least once a week had higher intakes of carbohydrates and fats (total, saturated, monounsaturated, and polyunsaturated). They also had lower serum folate levels compared to those who drank less frequently (≤ 4 drinks, once a month or less). In Spain, Escrivá-Martínez et al. (2023) observed a direct relationship between binge eating, high fat consumption, and alcohol abuse.

It is well established that diet quality and dietary factors play a key role in the prevention of non-communicable chronic diseases ( Global Burden of Disease et al., 2019; Laudisi et al., 2019). High UPF consumption adversely affects nutritional status ( Baroni et al., 2018), making it essential to maintain a healthy, balanced, and varied diet that is sustainable over time and contributes to healthy ageing.

This study presents some limitations that should be considered when interpreting the results. Regarding methodological aspects, the study employed a cross-sectional design, which prevents the establishment of causal relationships. In addition, dietary intake was estimated using a self-reported 24-hour recall questionnaire, which may be subject to recall bias; however, measures were taken to minimise this effect, including expert validation and pilot testing. Finally, the selection of products and participants was conducted in urban contexts using convenience sampling, which may limit the generalisability of the findings to other populations, such as rural or non-university youth.

### Ethical considerations

This study was conducted in accordance with the Code of Ethics guidelines established by César Vallejo University and the provisions of Rector’s Resolution No. 0126-2017-UCV. This study was approved by the Institutional Research Ethics Committee of Universidad César Vallejo (Approval ID: 0020-2023/CEI-FIA-DOC). Informed consent was obtained voluntarily and in written form via an initial question embedded in the online questionnaire, which allowed individuals to either confirm or decline their participation. Only those who provided explicit consent were permitted to proceed.

## Data availability

### Underlying data

Zenodo: Database: Ultra-Processed Food Consumption, Additive Co-occurrence, and Associated Risk Factors Among University Students in Peru” available from: DOI: https://doi.org/10.5281/zenodo.15678467 ( Guillén and Saenz, 2025).

This project contains the following underlying data:

• Consumption Patterns UPF Database.sav
• Food Additives UPF Database.sav

Data are available under the terms of the Creative Commons Attribution 4.0 International (CC-BY 4.0).

### Extended data

Zenodo: Database: Ultra-Processed Food Consumption, Additive Co-occurrence, and Associated Risk Factors Among University Students in Peru, https://doi.org/10.5281/zenodo.15939817 ( Guillén and Saenz, 2025).

This project contains the following underlying data:

• Extended data_Informed consent UPF.pdf
• Extended data_Questionnaire UPF.pdf

Data are available under the terms of the Creative Commons Attribution 4.0 International (CC-BY 4.0).
