# Supplementary material for: Consumo de alimentos ultraprocesados, coexistencia de aditivos y factores de riesgo asociados entre estudiantes universitarios en Perú
Source: F1000Res. 2026 Oct 22;14:854. Originally published 2025 Sep 2. [Version 3] doi: 10.12688/f1000research.166729.3 (PMC13695710; doi:10.12688/f1000research.166729.3)
Supplement: Peer review history

# Reviewer report: Reviewer response for version 3

**DOI:** 10.5256/f1000research.212552.r532767

**Published:** 2026-10-24

**Authors:** Juan García García

**Affiliations:** 1 Department of Public Health, Universidad Nacional Autónoma de México, Mexico City, Mexico

I am grateful for the replies to the comments made previously.

 For my part, I consider them to be satisfactory.

 Greetings

Is the work clearly and accurately presented and does it cite the current literature?

Yes

If applicable, is the statistical analysis and its interpretation appropriate?

Yes

Are all the source data underlying the results available to ensure full reproducibility?

Yes

Is the study design appropriate and is the work technically sound?

Yes

Are the conclusions drawn adequately supported by the results?

Yes

Are sufficient details of methods and analysis provided to allow replication by others?

Partly

Reviewer Expertise:

NA

I confirm that I have read this submission and believe that I have an appropriate level of expertise to confirm that it is of an acceptable scientific standard.

# Reviewer report: Reviewer response for version 2

**DOI:** 10.5256/f1000research.195530.r521700

**Published:** 2026-10-05

**Authors:** Juan García García

**Affiliations:** 1 Department of Public Health, Universidad Nacional Autónoma de México, Mexico City, Mexico

I find the work very interesting and addresses a growing public health problem, particularly in young people.

 I have some observations regarding the methods of selection of the participants and the products selected.

 Some of them, as they appear, have already been pointed out previously.

 The documents indicate that they used random sampling to choose the population addressed, but it is not clear where it was chosen from, and what type of probability sampling they used.

 The convenience sampling of the products may have depended on who or who carried out the choice of the products analyzed.

 An aspect out of control for the researchers is the fact that manufacturers declare all the additives used.

 I have the impression that the titles of the independent and dependent variables are inverted.

 If the information on the points indicated is expanded, I consider the article approved.

Is the work clearly and accurately presented and does it cite the current literature?

Yes

If applicable, is the statistical analysis and its interpretation appropriate?

Yes

Are all the source data underlying the results available to ensure full reproducibility?

Yes

Is the study design appropriate and is the work technically sound?

Yes

Are the conclusions drawn adequately supported by the results?

Yes

Are sufficient details of methods and analysis provided to allow replication by others?

Partly

Reviewer Expertise:

Epidemiología

I confirm that I have read this submission and believe that I have an appropriate level of expertise to confirm that it is of an acceptable scientific standard, however I have significant reservations, as outlined above.

# Response

**Published:** 2026-10-10

**Authors:** JHOSELINE STAYCE GUILLEN SANCHEZ

**Affiliations:** TRUJILLO, Cesar Vallejo University, Trujillo, La Libertad, Peru

We sincerely thank the reviewer for the positive comments regarding our study and for the valuable suggestions provided. The observations have been raised.

**The documents indicate that random sampling was used to select the study population; however, it is unclear where the sample was drawn from or what type of probability sampling was employed. **R: Thank you for your comments. Simple random sampling was employed, using the list of eligible students from various universities based on pre-established inclusion criteria as the sampling frame. Participants were selected using random numbers and subsequently invited to complete the digital questionnaire. This has been clarified in the methodology section.

**The convenience sampling of products may have depended on the individuals who selected the items for analysis. **R: Thank you for your comments. We acknowledge that convenience sampling may be influenced by the individuals responsible for selecting the products. However, we wish to clarify that the selection of ultra-processed food categories—including beverages, cookies, and other products was guided by previously published studies on food consumption in Peru (Mamani Urrutia et al., 2021; Ancka et al., 2022), rather than solely by the researchers' personal preferences. Furthermore, the specific products within these categories were selected based on their availability in commercial outlets.

**One aspect beyond the researchers' control is whether manufacturers disclose all the additives used. **R: Thank you for your comments. We agree that the declaration of food additives on product labels is a factor beyond the researchers' control. Therefore, this limitation has been incorporated into the analysis of food additive co-occurrence.

**I have the impression that the names of the independent and dependent variables have been swapped.**R. Thank you for your comments. The section on associated risk factors in the methodology has been reviewed and corrected.

 We greatly appreciate the time and effort devoted to reviewing our work.

 Sincerely,

# Reviewer report: Reviewer response for version 2

**DOI:** 10.5256/f1000research.195530.r458576

**Published:** 2026-02-27

**Authors:** Zheshen HAN

**Affiliations:** 1 The University of Hong Kong Li Ka Shing Faculty of Medicine (Ringgold ID: 71020, Hong Kong, Hong Kong

The author has addressed most of my concerns. While it's still mainly a descriptive study, I think it's okay for indexing now.

Is the work clearly and accurately presented and does it cite the current literature?

Yes

If applicable, is the statistical analysis and its interpretation appropriate?

Yes

Are all the source data underlying the results available to ensure full reproducibility?

Yes

Is the study design appropriate and is the work technically sound?

Yes

Are the conclusions drawn adequately supported by the results?

Partly

Are sufficient details of methods and analysis provided to allow replication by others?

Yes

Reviewer Expertise:

Epidemiology

I confirm that I have read this submission and believe that I have an appropriate level of expertise to confirm that it is of an acceptable scientific standard.

# Reviewer report: Reviewer response for version 1

**DOI:** 10.5256/f1000research.183763.r430860

**Published:** 2025-12-11

**Authors:** Zheshen HAN

**Affiliations:** 1 The University of Hong Kong Li Ka Shing Faculty of Medicine (Ringgold ID: 71020, Hong Kong, Hong Kong

1. The study tackles a pressing public health concern, the rising UPF consumption in Peru, linked to non-communicable diseases. It aligns with global concerns and fills a gap in data on Generation Z. The focus on additive co-occurrence adds novelty.

 2. This study combines food science with epidemiological study methods, providing a holistic view. The use of dendrograms for clustering is innovative for visualizing additive patterns.

 3. However, the study population (385 students from one university) may not represent all Peruvian youth, particularly rural or non-university populations. Product selection (500 from major chains) lacks details on sampling strategy (e.g., random or convenience), potentially biasing towards urban-preferred items.

 4. The study relies on 24-hour self-reported recall, prone to recall bias and under-/overestimation, and lacks further validation besides self-reported recall.

 5. Discussion overlooks limitations (e.g., bias, causality) and overstates conclusions ("excessive consumption... is significantly associated with a direct risk factor, alcohol consumption" implies causation).

 6. The author did not mention the response rate or non-response bias. Additive analysis lacks dose-response analysis.

 Revisions Suggested:

 7. The authors should detail product selection criteria (e.g., stratified by category/region) and survey administration.

 8. The authors should include multivariate regression with confounders (BMI, income, socioeconomic status, if applicable). The authors could add sensitivity analyses for recall bias. 

 9. The authors should add a detailed Limitation section (as limitations mentioned above), compare with other literature study results, and propose a mechanism hypothesis based on the study results.

Is the work clearly and accurately presented and does it cite the current literature?

Yes

If applicable, is the statistical analysis and its interpretation appropriate?

Yes

Are all the source data underlying the results available to ensure full reproducibility?

Yes

Is the study design appropriate and is the work technically sound?

Yes

Are the conclusions drawn adequately supported by the results?

Partly

Are sufficient details of methods and analysis provided to allow replication by others?

Yes

Reviewer Expertise:

Epidemiology

I confirm that I have read this submission and believe that I have an appropriate level of expertise to confirm that it is of an acceptable scientific standard, however I have significant reservations, as outlined above.

# Response

**Published:** 2026-01-20

**Authors:** JHOSELINE STAYCE GUILLEN SANCHEZ

**Affiliations:** TRUJILLO, Cesar Vallejo University, Trujillo, La Libertad, Peru

**The study tackles a pressing public health concern, the rising UPF consumption in Peru, linked to non-communicable diseases. It aligns with global concerns and fills a gap in data on Generation Z. The focus on additive co-occurrence adds novelty.**

**Response:** Thank you for your comments. We appreciate the recognition of the novel focus on additive co-occurrence. These remarks motivate us to continue advancing research in this highly relevant area.

**This study combines food science with epidemiological study methods, providing a holistic view. The use of dendrograms for clustering is innovative for visualizing additive patterns.**

**Response**: Thank you for your comments. We appreciate the recognition of the use of dendrograms as an innovative approach to visualise additive clustering patterns.

**However, the study population (385 students from one university) may not represent all Peruvian youth, particularly rural or non-university populations. Product selection (500 from major chains) lacks details on sampling strategy (e.g., random or convenience), potentially biasing towards urban-preferred items.**

**Response**: Thank you for your comments. The methodology now specifies that a non-probability convenience sampling approach was used, based on accessibility to formal urban retail outlets. It is also acknowledged that this strategy may introduce bias and limit representativeness in relation to other contexts, such as rural areas or informal markets. This observation has been addressed and can be found on page 3.

**The study relies on 24-hour self-reported recall, prone to recall bias and under-/overestimation, and lacks further validation besides self-reported recall.**

**Response**: Thank you for your comments. While we acknowledge that self-reported data may be subject to recall bias and estimation errors, this instrument was selected due to its widespread use in large-scale public health studies, given its efficiency in estimating recent food intake. In our study, it was validated by experts in public health, nutrition, and food technology, and was pilot-tested to ensure clarity and consistency, which helped minimise such effects. This reflection has been incorporated into the methodology section of the manuscript, highlighting both the limitations and strengths of the instrument used. This observation has been addressed and can be found on page 4.

**Discussion overlooks limitations (e.g., bias, causality) and overstates conclusions ("excessive consumption... is significantly associated with a direct risk factor, alcohol consumption" implies causation).**

**Response**: Thank you for your comments. This observation has been addressed and can be found on pages 1 and 8.

**The author did not mention the response rate or non-response bias. Additive analysis lacks dose-response analysis.**

**Response**: Thank you for your comments. All 385 questionnaires were completed; therefore, the response rate was 100%. Nevertheless, it should be noted that consumption percentages were calculated based on the valid responses within each product group, as not all participants answered all categories. This clarification was added as a note to the table and methodology (pages 5 and 15).

 Finally, a dose - response analysis of food additives was not conducted, as the aim of the study was to describe their presence and co-occurrence rather than individual exposure.

**The authors should detail product selection criteria (e.g., stratified by category/region) and survey administration.**

**Response**: Thank you for your comments. Additional details have been included in the methodology section. Products were selected using a convenience sampling approach, based on their accessibility and availability in urban supermarkets, and were classified into five main categories. Regarding the survey, it was self-administered and conducted in person within university facilities, under the supervision of the research team.

**The authors should include multivariate regression with confounders (BMI, income,**

**socioeconomic status, if applicable). The authors could add sensitivity analyses for recall bias.**

**Response**: Thank you for your comments. An ordinal logistic regression was incorporated, including multiple independent (predictor) variables such as age, sex, exercise modality, physical activity, place of eating, alcohol consumption, and tobacco use. This allows observation of the effect of each factor adjusted for the others (table 4). BMI, income, and socioeconomic status were not included because they were not collected and were not part of the original objectives or design. A prognostic matrix was also included to complement the regression and demonstrate the model's performance (table 5).

**The authors should add a detailed Limitation section (as limitations mentioned above), compare with other literature study results, and propose a mechanism hypothesis based on the study results.**

**Response**: Thank you for your comments. Limitations were added as the final paragraph within the discussion section, and a mechanism hypothesis was proposed based on the multivariate analysis and the predictive classification matrix.
